# Temporal intraspecific trait variability drives responses of functional diversity to interannual aridity variation in grasslands

**DOI:** 10.1002/ece3.5156

**Published:** 2019-04-12

**Authors:** Huiying Chen, Yongmei Huang, Kejian He, Yu Qi, Engui Li, Zhiyun Jiang, Zhilu Sheng, Xiaoyan Li

**Affiliations:** ^1^ Faculty of Geographical Science, State Key Laboratory of Earth Surface Processes and Resource Ecology, School of Natural Resources Beijing Normal University Beijing China; ^2^ School of Resource Environment and Earth Science Yunnan University Kunming China; ^3^ Inner Mongolia Environment Sciences Academy Hohhot China; ^4^ School of Geography South China Normal University Guangzhou China

**Keywords:** aridity, community‐weighted means, functional dispersion, functional traits

## Abstract

Interannual climate variation alters functional diversity through intraspecific trait variability and species turnover. We examined these diversity elements in three types of grasslands in northern China, including two temperate steppes and an alpine meadow. We evaluated the differences in community‐weighted means (CWM) of plant traits and functional dispersion (FDis) between 2 years with contrasting aridity in the growing season. Four traits were measured: specific leaf area (SLA), leaf dry matter content (LDMC), leaf nitrogen concentration (LNC), and the maximum plant height (H). CWM for SLA of the alpine meadow increased in the dry year while that of the temperate steppe in Qinghai showed opposing trends. CWM of LDMC in two temperate steppes became higher and CWM of LNC in all grasslands became lower in the dry year. Compared with the wet year, FDis of LDMC in the alpine meadow and FDis of LNC in the temperate steppe in Qinghai decreased in the dry year. FDis of H was higher in the dry year for two temperate steppes. Only in the temperate steppe in Qinghai did the multi‐FDis of all traits experience a significant increase in the dry year. Most of the changes in CWM and FDis between 2 years were explained by intraspecific trait variation rather than shifts in species composition. This study highlights that temporal intraspecific trait variation contributes to functional responses to environmental changes. Our results also suggest it would be necessary to consider habitat types when modeling ecosystem responses to climate changes, as different grasslands showed different response patterns.

## 1 INTRODUCTION

The interactions between plants and environment have long been of interest in ecological research (Wright et al., 2004). In the context of global climate change, extreme events are forecasted to occur more frequently and interannual climate variation is considerable (Easterling et al., 2000; Orlowsky & Seneviratne, 2011). Previous studies suggest that climate variability has great impacts on species richness, community productivity, and stability (Gherardi & Sala, 2015; Pérez‐Ramos et al., 2017; Zhang et al., 2018). However, the mechanisms of how plant communities respond to climate variation are yet to be fully understood. Plant functional traits are emerging as tools to explain these mechanisms as plants adjust to environmental variation by changing their traits (Cornelissen et al., 2003; Lavorel & Garnier, 2002). Scaling up to the community level, functional diversity appears to be a powerful indicator of community dynamics and assembly process (Kuiters, Kramer, Van der Hagen, & Schaminee, 2009; Petchey & Gaston, 2002), and it may affect the ecosystem stability indirectly (Cantarel, Bloor, & Soussana, 2013). Combining analyses of functional responses at both the species and community level will improve predictions about the effects of climate changes on vegetation (Holdaway, Richardson, Dickie, Peltzer, & Coomes, 2011; Katabuchi et al., 2017).

In a broad sense, functional diversity can be described using the community‐weighted means (CWM) of traits and the dispersion of traits in a community (Díaz et al., 2007; Garnier et al., 2004; Lavorel et al., 2008). The latter component can be expressed by various indices, among which Rao's quadratic entropy (Botta‐Dukat, 2005), functional dispersion (Laliberté & Legendre, 2010), and functional divergence (Villéger, Mason, & Mouillot, 2008) are widely used. Both the mean and dispersion of traits are influenced by environmental factors such as climate, land‐use regime, or biotic interactions (Carmona, Mason, Azcarate, & Peco, 2015; Pescador, Sierra‐Almeida, Torres, & Escudero, 2016; Schellenberger Costa et al., 2017). Environmental filters restrict the range of traits (Díaz, Cabido, & Casanoves, 1998), and a limiting similarity of coexisting species may occur due to fine‐scale niche partitioning (Cornwell & Ackerly, 2009; Muscarella & Uriarte, 2016). Therefore, the functional diversity of a community is the result of habitat filtering and/ or limiting similarity (Garnier & Navas, 2012).

Among all environmental factors, water is essential to plants. Therefore, aridity may restrict plant communities and lead to distinct functional composition (Nunes et al., 2017). The effects of aridity on functional diversity may vary between functional traits. For example, lower community mean specific leaf area (SLA) and higher leaf dry matter content (LDMC) were found to be associated with extreme drought (Jung et al., 2014), indicating that plants with conservative resource‐use strategies were favored (Costa‐Saura, Martinez‐Vilalta, Trabucco, Spano, & Mereu, 2016; Pérez‐Ramos et al., 2017). With increasing aridity, communities shifted to species with higher leaf nitrogen concentrations (LNC) in an arid steppe (Frenette‐Dussault, Shipley, Leger, Meziane, & Hingrat, 2012). Also, CWM of plant height decreased along spatial aridity gradients, as shorter plants faced less risk of cavitation under drought conditions (Gross et al., 2013; Nunes et al., 2017).

In addition, aridity may also influence functional dispersion (FDis). Higher aridity is associated with lower FDis, indicating species sharing similar functional traits adapted to aridity (Nunes et al., 2017). At dryer sites, harsher conditions may filter out species with nonviable strategies, resulting in lower FDis (Costa‐Saura, Trabucco, Spano, & Mereu, 2017; Dwyer & Laughlin, 2017). On the contrary, plants are found to develop different adaptations for some traits to survive in aridity, resulting in higher FDis (Stubbs & Wilson, 2004).

Above functional responses to environmental changes can occur via two processes. One is species turnover (regarding both changes in species identities and relative abundances) and the other is intraspecific trait variability due to plasticity and different genotypic compositions (Albert, Grassein, Schurr, Vieilledent, & Violle, 2011; de Bello et al., 2011). Disentangling the two processes can help us understand how communities respond to climate variation (de Bello et al., 2011) and the mechanisms of plant community assembly (Zuo et al., 2017). The relative contributions of them depend on the time or space scales of experiments (Volf et al., 2016). A significant contribution of species turnover often occurs over relatively long timescales, while large importance of intraspecific trait variability may occur relatively rapidly (Lajoie & Vellend, 2018). Though intraspecific variability in traits occurs spatially and temporally (Albert et al., 2010; Turner, Schulze, Nicolle, Schumacher, & Kuhlmann, 2008), less attention is paid to the temporal dimension since traits are usually sampled only once in a community (Violle et al., 2012). Besides spatial gradients, interannual aridity variation is also common and may influence functional diversity through changes in plant community composition (Carmona et al., 2015; Pérez‐Ramos et al., 2017). Few studies have taken temporal intraspecific trait variability into account when evaluating changes in functional diversity in response to climatically different years, but see Dwyer, Hobbs, and Mayfield (2014). Overall, it remains unclear how interannual climate aridity influences CWM and FDis via intraspecific trait variation and species turnover.

We select one alpine meadow and two types of temperate steppes located in northern China, which are both widespread types of grasslands in the Eurasian continent (Dixon, Faber‐Langendoen, Josse, Morrison, & Loucks, 2014). Interannual climate variation has been considerable during the past thirty years in the three grasslands (Gao, Li, Leung, Chen, & Xu, 2015; Tong, Zhang, & Bao, 2017). We measured four key functional traits and aboveground biomass of species in the three grasslands over 2 years with contrasting aridity. Our main objective is to quantify the relative contributions of intraspecific trait variation and species turnover to changes in CWM and FDis across years in each grassland system. Specifically, we hypothesized that (a) CWM and FDis of traits vary between the dry year and the wet year, with different patterns for different traits; (b) intraspecific trait variability contributes more than species turnover does to interannual changes in functional diversity over the wet year and the dry year.

## 2 METHODS

### 2.1 Study sites and sampling time

**Figure 1:**
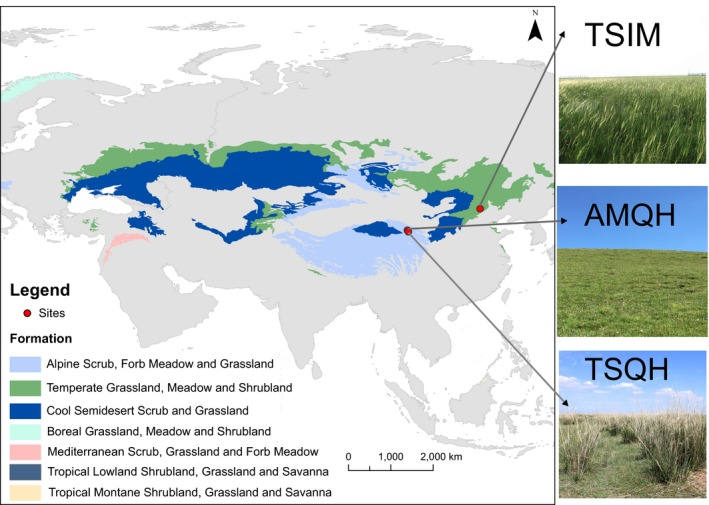
Locations and landscapes of three grasslands (AMQH, TSQH, and TSIM) on the map of world grassland types (Dixon et al., 2014)

This study was conducted in three grasslands in northern China (Figure 1). One was a *Stipa sareptana* var. *krylovii* steppe on the Inner Mongolian plateau, a typical temperate steppe (TSIM). The other two grasslands were both located in Qinghai Lake watershed on the Qinghai–Tibetan plateau, including a temperate steppe dominated by *Achnatherum splendens* (TSQH) and an alpine meadow with *Kobresia pygmaea* as the dominant species (AMQH). The three grasslands all have continental climates with a rainy, warm summer and a dry, cold winter, but their microenvironmental conditions were quite different (Table 1). For each type of grassland, we selected three sites with a minimum distance between adjacent sites of 1 km to make sure that all sites were independent.

**Table 1:** Environmental characteristics of the three grasslands

	AMQH	TSQH	TSIM
Elevation (m a.s.l.)	3,530	3,168	1,450
Longitude	100.06 E	100.25 E	115.49 E
Latitude	37.56 N	37.24 N	42.11 N
Vegetation type	Alpine meadow	Temperate steppe	Temperate steppe
Slope and aspect	15° southwest	Flat	Flat
Soil type	Alpine meadow soil	Chestnut soil	Light chestnut soil
Management	Fenced and lightly grazed in spring	Fenced and lightly grazed in winter	Fenced and no grazing

According to long‐term records of the nearest National Weather Station (Taibus Banner), TSIM has a mean annual temperature of 1.6°C and annual precipitation of 400 mm (He et al., 2016). The only one National Weather Station near AMQH and TSQH has the mean annual temperature of 0.1°C and annual precipitation of 389.4 mm (Zhang, Li, Zhao, & Huang, 2016). As AMQH is located on hillsides in the middle reaches of Shaliu River, it has a wetter and colder climate than the lower reaches where TSQH is located. In this case, we built two small weather stations in June 2012 at one site of AMQH and one site of TSQH, respectively, to record real‐time precipitation and air temperature automatically (more details about the measurements of meteorological parameters can be found in Zhang et al., 2016). For TSIM, we used climatic data from the Taibus Banner Weather Station. The growing season of the three grasslands usually starts in late April and ends in late September, lasting for about 5 months. As such, we did all field measurements in late August in two years when the plant community reached peak biomass (in 2013 and 2014 for AMQH and TSQH and in 2012 and 2014 for TSIM). We calculated the ratio of potential evapotranspiration (PET) to precipitation as the aridity index, following the formula PET (mm) = 58.93 * (number of days in one period/ number of days in one year) * mean bio‐temperature (Holdridge, 1959). To make a more accurate description of aridity, we calculated the aridity index for the period from the beginning of the growing season to the day we sampled instead of for the whole year. A higher index indicates more arid environment, and the two sampling years could be classified as a wet year and a dry year (Table 2). Long‐term records from the nearest stations also supported the classification. In the dry year, the aridity index for the same period was 123.13% (Gangcha Station near AMQH and TSQH) and 117.88% (Tabus Banner Station near TSIM) of the 5‐year average value, while in the wet year the ratios were 79.90% and 83.23%, respectively. Compared to the wet year, all sites experienced lower precipitation and higher temperature in the dry year (Table 2).

**Table 2:** Aridity index, precipitation, and temperature during the growing season in the three grasslands, which were calculated for the period of 4 months before the sampling date (late August)

Site	Year		Aridity index		Precipitation (mm)		Temperature (°C)	
	Wet	Dry	Wet	Dry	Wet	Dry	Wet	Dry
AMQH	2014	2013	0.31	0.61	323.50	267.80	5.02	8.18
TSQH	2014	2013	0.63	1.32	260.29	154.94	8.23	10.28
TSIM	2012	2014	0.94	1.34	278.20	203.40	13.35	13.83

Note

Data for AMQH and TSQH are from the weather station we installed, while data of TSIM are from the nearest National Weather Station.

### 2.2 Vegetation surveys

We assessed the community structure by the relative aboveground biomass of species, using three sampling quadrats at each site. In the first year, we sampled randomly in each site and marked the quadrat positions by short sticks. To avoid the effects of mowing, the locations of quadrats changed but slightly in the second year. As TSQH comprised two layers (Figure 1), a taller layer of *A. splendens* tussocks and a lower layer of other short plants, we measured the aboveground biomass in several steps. First, quadrats of 5 * 5 m were used to measure coverage and height of every *A. splendens* tussock. Then, several typical tussocks were harvested and dried to build an equation between the volume of a tussock and the aboveground biomass, which could be used to estimate the biomass of all tussocks. Then, we set smaller quadrats of 1 * 1 m inside the larger quadrats to record other plants. All aboveground parts of short plants were harvested and sorted into species and then dried for 24 hr to a constant weight. Total aboveground biomass of a 5 * 5 m quadrat (*B_t_*, g) can be calculated as:

$$B_{t}=\left(A_{t}-A_{\mathrm{Asp}}\right)*B_{o}+B_{\mathrm{Asp}}$$

*A_t_* is the area of the quadrat (5 * 5 m), *A_Asp_* (m^2^) is the sum of projected area of all *A. splendens* tussocks, *B_o_* (g/m^2^) is the aboveground biomass of other plants in the 1 * 1 m quadrat, and *B_Asp_* (g) is the sum of the aboveground biomass of all *A. splendens* tussocks. Then, the relative biomass of *A. splendens* (*p_Asp_*) and other species (*p_i_*) in a community can be calculated as follows:

$$p_{\mathrm{Asp}}=B_{\mathrm{Asp}}/B_{t}$$

$$p_{i}=\left(A_{t}-A_{\mathrm{Asp}}\right)*B_{i}/B_{t}$$

*B_i_*(g/m^2^) is the aboveground biomass of species *i* in the 1 * 1 m quadrat. For TSIM and AMQH, we used quadrats of 1 * 1 m to do the survey and also calculated the relative biomass of each species. The three grasslands shared no species at the same time.

### 2.3 Trait measurements

We measured specific leaf area (SLA, leaf area per unit of dry leaf mass, m^2^/kg), leaf dry matter content (LDMC, leaf dry mass per unit of water‐saturated fresh mass, g/kg), leaf nitrogen concentration based on mass (LNC, g/kg), and maximum plant height (H, cm) of the common species across nine sites in the three grasslands, which made up to 94% on average (between 83% and 100%) of the community aboveground biomass (Pakeman & Quested, 2007). We recorded 81 species in vegetation surveys in 2 years and measured four traits on the most common 31 species in the dry year and the wet year. All materials were collected from robust, well‐grown plants across populations using standard protocols (Cornelissen et al., 2003; Pérez‐Harguindeguy et al., 2013). For *Stipa aliena* and *Tibetia himalaicain* in AMQH, their traits were measured again in 2015 because data in 2013 and 2014 were lost. They accounted for 12.04% and 12.24% of the community aboveground biomass in 2013 and 2014. The aridity index is 0.38 for the growing season in 2015 at AMQH, close to the value 0.30 in the wet year.

### 2.4 Functional diversity calculation

We calculated CWM for each trait, which was the community‐level mean of trait values weighted by the relative abundance of each species (Garnier et al., 2004). We used relative aboveground biomass of species to represent relative abundance. Besides CWM, we also calculated functional dispersion (FDis) to describe trait dispersion (Laliberté & Legendre, 2010; Schleicher, Peppler‐Lisbach, & Kleyer, 2011). FDis is the average distance of individual species to the centroid of all species in the community trait space, and it is a multidimensional index based on multitrait space (Laliberté & Legendre, 2010). Changing trend of FDis may differ between traits in response to aridity variation. To catch if there are specific patterns, we also calculated FDis for every trait. We used the average trait value of all individuals of one species measured in each year to calculate CWM and FDis. These trait values are here called “specific” traits. All functional indices were calculated by the R package “FD” (Laliberté & Legendre, 2010; R Core Team, 2016).

### 2.5 Statistical analysis

We used linear mixed‐effects models to examine the effects of year, grassland type, and their interaction on CWM and FDis, with site as a random factor. The models were performed within the R package “lme4” (Bates, Machler, Bolker, & Walker, 2015). We also calculated *p*‐values in the R package “lmerTest” (Kuznetsova, Brockhoff, & Christensen, 2016) and obtained marginal *R* ^2^ and conditional *R* ^2^ with the R package “MuMIn” (Bartoń, 2016). For each grassland, ANOVA was applied for testing differences in CWM and FDis between the 2 years, as well as the community aboveground biomass. At the species level, we also compared traits of each species between the 2 years by ANOVA. We evaluated differences in functional diversity between grasslands in the same year by ANOVA, with post hoc Tukey test. All data were checked to fulfill the assumptions of normality and homogeneity of variance. Otherwise, the data were log‐transformed. ANOVA was conducted using IBM SPSS Statistics 22 (IBM Corp., NY, USA). Finally, differences in species composition (based on species relative biomass) between years were examined by permutational multivariate analysis of variance (PERMANOVA) with the “vegan” package in R (Oksanen et al., 2017). Bray–Curtis dissimilarity index was chosen to calculate the distance matrix.

We calculated the relative contribution of intraspecific trait variation and species turnover using the method based on a sum of squares decomposition that calculates CWM and FDis of “specific” traits and “fixed” traits (de Bello et al., 2011; Lepš, de Bello, Smilauer, & Dolezal, 2011). A “specific” trait for one species was the average trait value of individuals measured in the same type of grassland during the same year. A “fixed trait” for one species was the average of all values for one trait measured in 2 years in the same grassland. As a result, interannual changes in functional diversity based on the “fixed” trait were only caused by differences in species composition. Intraspecific trait variability across years can be represented by the differences in community metrics calculated using “fixed” and “specific” traits (de Bello et al., 2011; Lepš et al., 2011). Finally, the total sum of squares (SS‐specific) of the functional diversity variation related to interannual variation can be disentangled into “fixed” (SSfixed), “intraspecific” (SSintra), and “covariation” (SScov) effects (de Bello et al., 2011; Lepš et al., 2011). A positive covariation suggests that the dry year (or the wet year) selecting for certain dominant trait values may also influence trait plasticity in the same direction (Volf et al., 2016). The calculation was done using the R functions provided by Lepš et al. (2011).

## 3 RESULTS

### 3.1 Patterns in community‐weighted means (CWM) of traits

**Figure 2:**
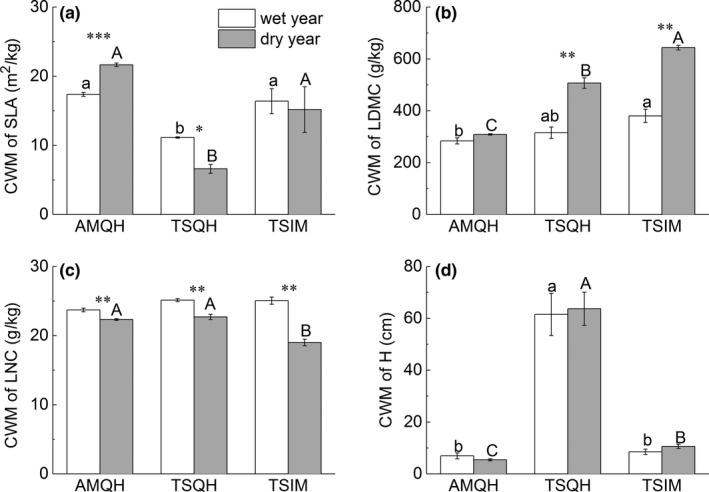
Community‐weighted mean values (CWM) of functional traits different traits (a: SLA; b: LDMC; c: LNC; d: H) of three grasslands between 2 years. Different letters indicate significant differences between grassland types (lower case for the wet year and uppercase for the dry year). Asterisks show significant differences between years for a given grassland type. Error bar was the standard error. *p < 0.05; **p < 0.01; ***p < 0.001

Except for plant height, all community mean traits differed significantly between years (Table 3; Figure 2). CWM of SLA in AMQH increased by more than 24% in the dry year compared with the wet year (*p* < 0.05, Figure 2a). In contrast, CWM of SLA in TSQH decreased by about 40% (*p* < 0.05, Figure 2a). CWM of LDMC in two temperate steppes both increased significantly in the dry year (*p* < 0.05, Figure 2b), while there was no difference for the alpine meadow (AMQH). CWM of LNC in all grasslands experienced a significant decline in the dry year (*p* < 0.05, Figure 2c).

**Table 3:** Results of the linear mixed‐effects models analyzing values of community‐weighted mean and functional dispersion for each functional trait

	Grassland type	Year	Type * year	Marginal *R* ^2^	Conditional *R* ^2^
	*F*	*F*	*F*		
Community‐weighted means (CWM)					
SLA	13.58**	0.60	16.42**	0.77	0.94
LDMC	80.11***	132.01***	25.81***	0.95	0.95
LNC	11.25**	157.64***	29.30***	0.92	0.94
H	109.34***	0.07	0.12	0.93	0.93
Functional dispersion (FDis)					
FDis‐SLA	1.66	0.01	1.47	0.27	0.39
FDis‐LDMC	216.90***	19.54***	68.69***	0.97	0.97
FDis‐LNC	9.18*	11.76*	14.57**	0.76	0.83
FDis‐H	656.06***	13.54**	14.10***	0.99	0.99
Multi‐FDis	243.80***	3.31	7.55*	0.97	0.97

Note

Grassland type, year, and their interaction were used as fixed factors. Site was included as a random factor.

* *p* < 0.05;

** *p* < 0.01;

*** *p* < 0.001.

Besides interannual differences in CWM, we also found large differences between grasslands in CWM for all traits (Table 3; Figure 2). Plants in TSQH had the lowest SLA and the highest plant height in both the dry year and the wet year (Figure 2a,d). There was no difference in community mean LDMC between the two temperate steppes in the wet year but in the dry year there were significant differences in CWM of LDMC among the three grasslands (*p* < 0.05, Figure 2b). In the dry year, CWM of LNC in TSIM was significantly lower than that in the other two grasslands (*p* < 0.05, Figure 2c), and in the wet year, there was no difference in LNC among different grasslands (Figure 2c).

### 3.2 Patterns in functional dispersion (FDis)

**Figure 3:**
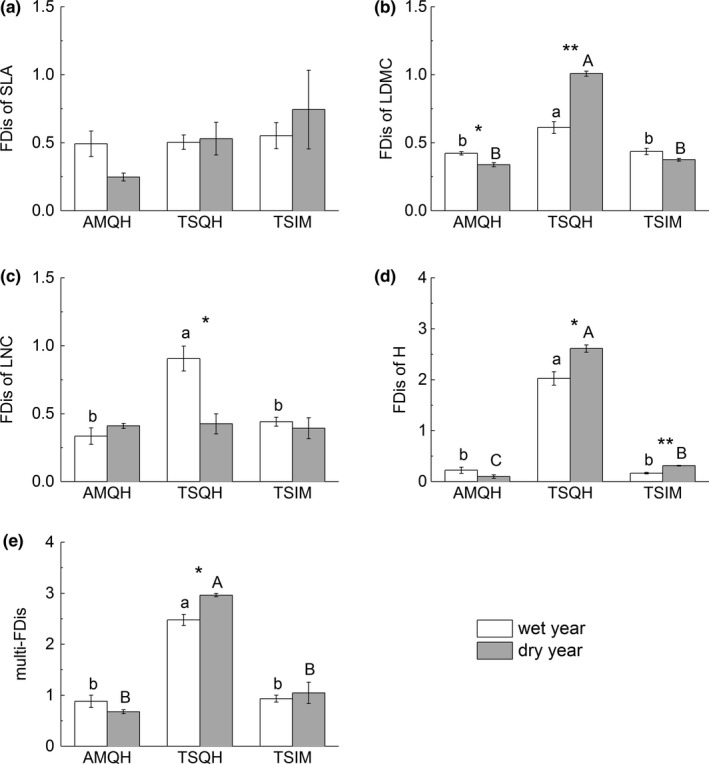
Functional dispersion (FDis) of different traits (a: SLA; b: LDMC; c: LNC; d: H; e: all traits) of three grasslands between 2 years. Different letters indicate significant differences between grassland types (lower case for the wet year and uppercase for the dry year). Asterisks show significant differences between years for a given grassland type. Error bar was the standard error. *p < 0.05; **p < 0.01

Temporal shifts in CWM of traits were accompanied by shifts in functional dispersion (Table 3; Figure 3). No significant change was detected in FDis of SLA, either among grasslands or between years (Table 3; Figure 3a). FDis of LDMC in AMQH was lower in the dry year than in the wet year (*p* < 0.05, Figure 3b). However, in TSQH FDis of LDMC in the dry year increased to over 1.6 times of that in the wet year (*p* < 0.05, Figure 3b). FDis of LNC in AMQH and TSIM were both constant across years while this index decreased by half in the dry year in TSQH (*p* < 0.05, Figure 3c). Plant height (H) in the two temperate steppes both became more divergent in the dry year (*p* < 0.05, Figure 3d). Multi‐FDis of all traits increased in TSQH in the dry year, but there was no significant difference at AMQH or TSIM between 2 years (Figure 3e). Grassland type also played an important role in functional dispersion (Table 3; Figure 3). In general, communities in TSQH had the highest FDis (*p* < 0.05, Figure 3).

### 3.3 Relative contributions of intraspecific trait variation and species turnover

**Figure 4:**
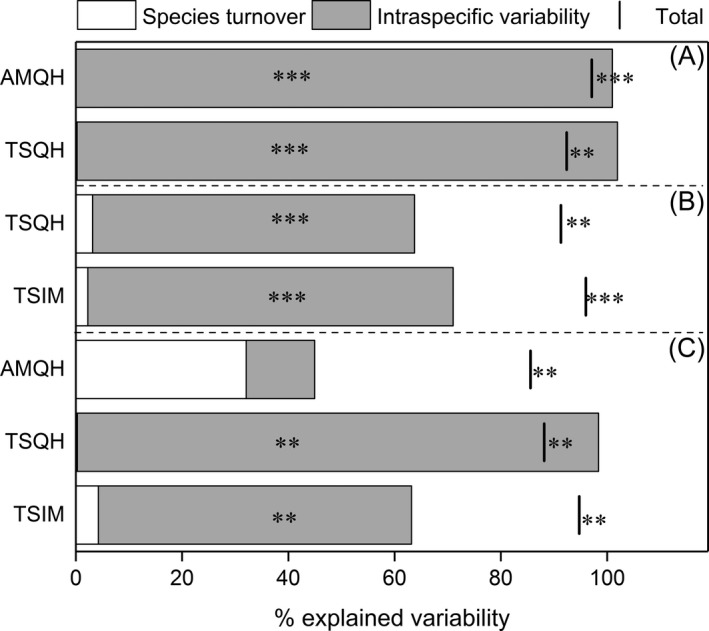
Decomposition of the total variations in community‐weighted means explained by years with contrasting aridity into intraspecific variability, species turnover, and covariation effects for (a), CWM‐SLA; (b), CWM‐LDMC; (c), CWM‐LNC. We operated the decomposition if there was a significant difference in CWM of a trait in a grassland between the dry year and the wet year. The interval between the bar and the top of the column represents the covariation effect; if the bar is on the right side the column, the covariation is positive; if the bar crosses the column, the covariation is negative. **p < 0.01; ***p < 0.001

**Figure 5:**
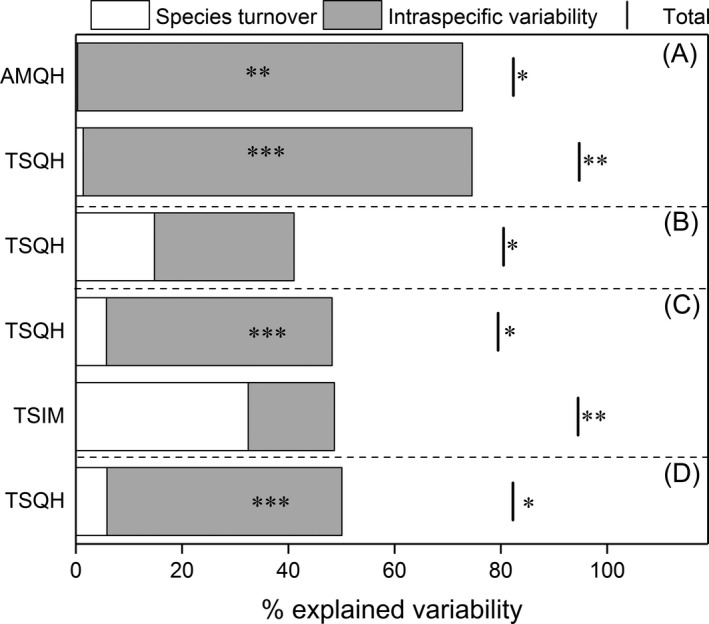
Decomposition of the total variability in functional dispersion of traits explained by years with contrasting aridity into intraspecific variability, species turnover, and covariation effects for (a), FDis‐LDMC; (b), FDis‐LNC; (c), FDis‐H; (d), multi‐FDis. We operated the decomposition if there was a significant difference in FDis of a trait in a grassland between the dry year and the wet year. The interval between the bar and the top of the column represents the covariation effect; if the bar is on the right side the column, the covariation is positive; if the bar crosses the column, the covariation is negative. *p < 0.05; **p < 0.01; ***p < 0.001

For all observed interannual differences in CWM of three leaf traits, intraspecific variability accounted for most of the total variation with only one exception of CWM‐LNC in AMQH (Figure 4), whereas species turnover contributed much less (Figure 4). At the same time, we found no significant differences either in aboveground biomass or the overall species composition between years in all grasslands (Table 4 and Supporting Information Table S1). The covariation between intraspecific variability and species turnover was slightly negative for changes in CWM of SLA in two grasslands in Qinghai, LNC in TSQH, and it was positive for others (Figure 4). Patterns were similar for FDis. The variability between years was mainly explained by intraspecific trait variability (except FDis for H in TSIM). All covariations of these two effects were positive for changes in functional dispersion between years, indicating similar effects of species turnover and intraspecific variability on the response of FDis (Figure 5).

**Table 4:** PERMANOVA results for compositional differences in the three grasslands between 2 years

	*df*	MS	*F*	*R* ^2^	*p*
AMQH					
Year	1	0.09	2.72	0.41	0.20
Residuals	4	0.03		0.59	
Total	5			1.00	
TSQH					
Year	1	0.09	1.49	0.27	0.30
Residuals	4	0.06		0.73	
Total	5			1.00	
TSIM					
Year	1	0.13	0.96	0.19	0.60
Residuals	4	0.13		0.81	
Total	5			1.00	

## 4 DISCUSSION

Our results demonstrate that interannual aridity variation drove considerable changes in CWM and FDis of traits in different grasslands. These two components of functional diversity had different patterns for different traits. For the same trait, we observed inconsistent responses of the three grasslands to interannual aridity variation, which may result from contrasting environmental conditions and different community structures. For most changes in functional diversity between the wet year and the dry year, intraspecific variability in traits over 2 years contributed more than species turnover.

### 4.1 CWM responses to interannual aridity variation

Community‐weighted means of three leaf traits varied over 2 years with contrasting aridity, but the changing directions differed among traits and grasslands. In the dry year, CWM of specific leaf area (SLA) decreased in one temperate steppe (TSQH) but increased in the alpine meadow (AMQH), associated with similar changes at the species level, respectively (Supporting Information Table S2). Consistent with the pattern observed in TSQH, previous studies at species and community levels both found that SLA decreased to cope with water scarcity (Costa‐Saura et al., 2017; Dwyer et al., 2014). The reverse responses of two grasslands may result from their different climatic conditions. AMQH had a relatively high soil water content both in the dry year and the wet year (Zhang & Li, 2017), so temperature instead of water would be the key limitation for community function there. In AMQH, the average temperature during the growing season in the dry year was 3.16°C higher than that in the wet year (Table 2). An experimental warming study for *K. pygmaea*, the dominant species in AMQH, increased SLA in response to an increase of 4.4°C in air temperature (Yang, Wang, Klanderud, & Yang, 2011). Higher SLA is usually associated with a higher relative growth rate (Díaz & Cabido, 1997).

Leaf dry matter content generally increases with lower water availability (Schob, Armas, Guler, Prieto, & Pugnaire, 2013). Aridity favored higher LDMC in two temperate steppes, which resulted in significant differences among grasslands in the dry year (Figure 2b). High LDMC indicates high investment in structural tissues, resulting in relatively tough leaves (Jung et al., 2014; Pérez‐Harguindeguy et al., 2013). Leaves with high dry matter contents are usually drought‐tolerant because they can keep turgor from decreasing sharply when soil water potential becomes lower (Pescador, de Bello, Valladares, & Escudero, 2015).

Leaf nitrogen content (LNC) has a close relationship with water‐use efficiency and photosynthetic capacity (Wright et al., 2004). We found all grasslands had lower CWM of LNC in the dry year. An experimental water addition study reported similar results (Sandel et al., 2010). As soil nitrogen content was reported to decline with aridity globally (Delgado‐Baquerizo et al., 2013) and LNC depends on the soil nitrogen content (Ghiloufi & Chaieb, 2015), aridity may lead to lower LNC. In contrast, CWM of LNC increased with aridity along a small aridity gradient in an arid steppe (Frenette‐Dussault et al., 2012). At the species level, LNC of plants increases with aridity globally (Wright et al., 2004). On the Qinghai–Tibetan plateau, the effect of annual precipitation was not significant for LNC (He et al., 2006). Overall, how LNC responds to aridity may be case‐specific, depending on the research scale or degree of aridity.

In our research, we found no significant difference in plant height over 2 years. Plant height is often considered to reflect the competitive ability (Frenette‐Dussault et al., 2012). Lower CWM of plant height was observed under drier conditions in Mediterranean drylands because annual plants were more abundant at drier sites (Nunes et al., 2017). In our case, height was rather constant because the fluctuations in aridity between years may not have been severe enough to cause significant changes in plant height. At the same time, species responded in different directions to aridity so that we could not find apparent changes at the community level (Supporting Information Table S2).

### 4.2 Aridity led to an increase in functional dispersion for some traits

Aridity did not necessarily decrease functional dispersion. FDis of different traits or in different grasslands had different responses to aridity. Only FDis of LNC in TSQH and FDis of LDMC in AMQH decreased in the dry year compared to the wet year. Previous work found FDis of SLA and some reproductive traits decreased with aridity in a Mediterranean dryland ecosystem (Nunes et al., 2017), indicating that communities occupy less trait space (Weigel, Blenckner, & Bonsdorff, 2016). Interestingly, four FDis metrics increased in the dry year in our study (Figure 3). Higher FDis reflects various and complementary strategies among species (Mouillot, Villeger, Scherer‐Lorenzen, & Mason, 2011), which may contribute to some ecosystem functions (Zhang, Wang, Kaplan, & Liu, 2015). Despite decreasing in resource availability, communities sometimes show trait divergence instead of convergence (Bernard‐Verdier et al., 2012).

TSQH experienced the most significant changes in FDis than other grasslands between the 2 years. TSQH had an obvious patchy structure, while AMQH and TSIM were homogeneous (Figure 1). Compared to interpatch zones, *A. splendens* patches had greater soil water availability (Jiang et al., 2017). This kind of community structure partly explained the highest functional dispersion among the three grasslands. Though species turnover accounted for small variability in FDis of LDMC and plant height in TSQH (Figure 5), TSQH changed from communities with one dominant species to communities with *Artemisia frigida* as the second dominant species in the dry year (Supporting Information Figure S1). This may explain the increase in FDis of traits in TSQH.

### 4.3 Mechanisms of functional diversity variation

In our study, changes in functional diversity between the dry year and the wet year were mainly driven by the intraspecific variability in traits. This result suggests that intraspecific variability buffer communities from strong species turnover through plastic adjustments to interannual aridity variation. In this way, intraspecific variability contributes to the short‐term stability of communities (Jung et al., 2014; Lloret, Escudero, Iriondo, Martínez‐Vilalta, & Valladares, 2012). This may partly explain the observed stable aboveground biomass of communities over the dry year and wet year.

For some studies based on controlled experiments, intraspecific variations in trait values also played a more important role than species turnover in response to certain abiotic factors, for example, nitrogen and water (Jung et al., 2014; Lü et al., 2018). However, opposite results were observed along an elevation gradient (Kichenin et al., 2013), which was mainly due to wide sampling of various vegetation types with quite different species compositions. Similar contrasting patterns were also found between short‐ and long‐term management on functional diversity (Volf et al., 2016). In the face of increasing aridity, changes may occur at the individual level in the first stage by altering traits, followed by changes in species composition at the community level, and finally some species will move out of the communities or new species will arrive (Sandel et al., 2010). Climate variation between the 2 years in this study represents short‐term influences and more changes in species composition are expected if aridity continues to change in the future.

Previous studies have evaluated the relationship between functional diversity and environment during a short or long period (Carmona et al., 2015; Kuiters et al., 2009), but they ignored the year‐specific trait data. Our results highlight that taking temporal intraspecific variations of traits into consideration may lead to different and interesting findings. Our results were observed in 2 years with contrasting aridity. In the future, a long‐term study on both CWM and FDis is required. Besides mature long‐term recording of plant communities and meteorological data, long‐term observations of plant functional traits are also necessary. When using trait data from global database like TRY or LEDA (Kattge et al., 2011; Kleyer et al., 2008), data from matching climatic conditions may produce more credible results. If traits measured in a wet year were used to calculate functional indices for communities in a dry year, conclusions might be misleading (Dwyer et al., 2014).

Different patterns of the three grasslands in response to aridity variation in our study were based on different community structures and environmental conditions. When modeling ecosystem responses to climate variation, vegetation type may not be neglected as different grasslands showed different response patterns. It should be noted that differences in aridity between 2 years were not exactly the same for three grasslands. In TSIM, the difference of aridity index between 2 years was the smallest among the three grasslands. Our results should be interpreted under this limitation in mind. Additionally, temporal intraspecific variability of two species in AMQH was not reflected as their traits were measured in the supplementary survey in 2015. As the year 2015 is closer to a wet year in AMQH, responses of functional diversity may be more significant to interannual climate variation than we reported.

## 5 CONCLUSION

Despite previous studies about the response of functional diversity to aridity, it is still unclear how interannual climate variation influences both community mean trait values and functional dispersion, especially with the temporal intraspecific variability in traits being included. Functional diversity of the four traits we studied showed various responses to interannual fluctuations in aridity, and these depended on the type of grassland. Thus, taking habitat types into consideration will improve the modeling of ecosystem responses to global climate changes. We also found that aridity did not reduce functional dispersion necessarily. This study highlights that intraspecific variations in plant traits across years are important for functional diversity to the changing environment. Our findings may provide evidence for predicting responses of plant communities to global climate changes.

## CONFLICT OF INTEREST

None declared.

## AUTHOR CONTRIBUTION

Y.M.H. and X.Y.L. designed the study. H.Y.C. and Y.M.H. developed the hypotheses. H.Y.C, K.J.H, Y.Q., and Z.Y.J. carried out the experiment. H.Y.C. analyzed the data with the help of E.G.L. and Z.L.S. H.Y.C. wrote the manuscript, and all authors contributed to revisions.

## Data Availability

Data available from the Dryad Digital Repository: https://doi.org/10.5061/dryad.5q66f0n.
