# Supplementary material for: Temporal intraspecific trait variability drives responses of functional diversity to interannual aridity variation in grasslands
Source: Ecol Evol. 2019 Apr 12;9(10):5731–42. doi: 10.1002/ece3.5156 (PMC6540671; doi:10.1002/ece3.5156)
Supplement: Supplementary file 1 [file ECE3-9-5731-s001.docx]

FIGURE S1. Traits and corresponding relative biomass of species in three grasslands. Relative biomass is the mean value among three sites in a grassland. Solid lines and dotted lines represent the average CWM of a trait in the wet year and the dry year respectively.

TABLE S1

Aboveground biomass of three grasslands between two years (average value± standard error). Results of ANOVA between two years are also shown.

| Grassland types | Aboveground biomass (g·m^-2^) | | p-value |
| --- | --- | --- | --- |
|  | Wet year | Dry year |  |
| AMQH | 156.65±12.45 | 182.74±7.20 | 0.14 |
| TSQH | 86.44±8.48 | 58.74±10.56 | 0.11 |
| TSIM | 127.95±7.24 | 115.68±10.03 | 0.38 |

Table S2

Plant traits measured in the wet year and the dry year in three grasslands. Significant differences in traits between two years are shown in red bold font (P<0.05). The word “null” means the species was not found in communities in this year, so no traits were measured.

|  |  | SLA  (m^2^ kg^-1^) | | LDMC  (g kg^-1^) | | LNC  (g kg^-1^) | | H  (cm) | |
| --- | --- | --- | --- | --- | --- | --- | --- | --- | --- |
|  | Species | wet | dry | wet | dry | wet | dry | wet | dry |
| A  M  Q  H | *Elymus nutans* | 17.77 | 15.66 | 405.56 | 478.22 | 24.80 | 21.57 | 20.18 | 26.58 |
|  | *Gentiana lawrencei* var. *farreri* | 18.30 | 17.10 | 204.76 | 226.87 | **20.00** | **18.40** | 6.25 | 9.67 |
|  | *Kobresia pygmaea* | **16.69** | **22.21** | 379.34 | 359.41 | **23.47** | **20.97** | 3.11 | 4.32 |
|  | *Polygonum viviparum* | **11.77** | **16.79** | 189.13 | 253.22 | 27.40 | 33.07 | 14.67 | 18.33 |
|  | *Potentilla saundersiana* | 15.19 | 19.57 | **221.61** | **358.88** | 21.63 | 25.25 | 5.67 | 5.50 |
|  | *Saussurea pulchra* | **13.36** | **17.17** | 142.84 | 134.65 | 21.33 | 21.50 | 5.25 | 4.17 |
|  | *Stipa aliena* | 37.29 | 37.29 | 226.40 | 226.40 | 20.93 | 20.93 | 18.75 | 18.75 |
|  | *Tibetia himalaica* | 23.75 | 23.75 | 246.37 | 246.37 | 26.57 | 26.57 | 5.33 | 5.33 |
|  | *Trollius pumilus* | 16.78 | null | 188.47 | null | 27.90 | null | 8.33 | null |
| T  S  Q  H | *Achnatherum splendens* | **8.03** | **2.33** | **202.07** | **288.75** | **28.73** | **21.73** | 97.33 | 116.00 |
|  | *Allium tanguticum* | 12.34 | 11.91 | 236.36 | 187.60 | **28.45** | **31.40** | 15.83 | 15.03 |
|  | *Artemisia frigida* | **10.03** | **7.14** | **466.09** | **716.04** | 23.33 | 25.20 | **7.69** | **12.10** |
|  | *Aster altaicus* | **17.11** | **9.26** | **359.17** | **638.75** | **25.60** | **28.67** | 11.00 | 13.47 |
|  | *Carex coriophora* | 9.52 | 7.14 | 687.17 | 747.30 | 13.10 | 13.50 | **14.00** | **9.50** |
|  | *Dracocephalum heterophyllum* | 16.29 | null | 309.29 | null | 27.37 | null | 11.13 | null |
|  | *Gentiana dahurica* | 9.26 | null | 344.86 | null | 23.00 | null | 2.25 | null |
|  | *Kobresia humilis* | **12.05** | **6.78** | **311.63** | **825.92** | **16.20** | **13.53** | **7.33** | **12.10** |
|  | *Poa faberi* var. *longifolia* | 13.87 | 14.73 | 650.00 | 683.33 | 16.80 | 15.77 | 15.00 | 9.90 |
|  | *Stipa purpurea* | **31.61** | **17.50** | **368.00** | **741.75** | **14.00** | **20.83** | 18.98 | 24.01 |
|  | *Stipa sareptana* var. *krylovii* | 30.26 | 32.25 | **290.00** | **820.72** | **15.30** | **21.80** | 30.00 | 31.00 |
| T  S  I  M | *Allium ramosum* | 16.16 | null | 148.84 | null | 30.59 | null | 14.00 | null |
|  | *Allium tenuissimum* | 21.32 | 13.47 | **99.21** | **294.67** | **30.41** | **22.70** | **10.97** | **6.61** |
|  | *Androsace longifolia* | 14.29 | 12.03 | **235.48** | **419.08** | **27.04** | **21.68** | 3.23 | 4.00 |
|  | *Artemisia frigida* | 16.13 | 8.80 | 490.68 | 574.36 | 23.79 | 20.85 | **9.44** | **4.00** |
|  | *Artemisia capillaris* | 11.14 | null | 200.00 | null | 23.17 | null | 16.40 | null |
|  | *Cleistogenes squarrosa* | 7.06 | 12.58 | **281.25** | **801.27** | 21.49 | 20.30 | **3.66** | **6.83** |
|  | *Convolvulus ammannii* | 14.33 | 11.43 | **292.01** | **547.29** | **28.18** | **21.33** | 5.02 | 5.44 |
|  | *Cymbaria dahurica* | 10.78 | 9.51 | **432.43** | **664.38** | 18.65 | 18.60 | **6.17** | **8.00** |
|  | *Dracocephalum rupestre* | 8.49 | null | 467.72 | null | 24.30 | null | 5.37 | null |
|  | *Leymus chinensis* | 8.81 | 7.28 | **250.00** | **733.35** | 19.40 | 18.40 | 17.00 | 17.83 |
|  | *Potentilla bifurca* var. *major* | 10.50 | 7.80 | **465.01** | **658.05** | **24.20** | **18.77** | **6.33** | **3.50** |
|  | *Salsola collina* | 2.34 | null | 173.68 | null | 31.30 | null | 5.34 | null |
|  | *Stipa sareptana* var. *krylovii* | 29.17 | 29.88 | **519.12** | **749.57** | **22.88** | **15.37** | 15.47 | 19.11 |
